# Spectral Reflectance Reconstruction of Organ Tissue Based on Metameric Black and Lattice Regression

**DOI:** 10.3390/s22239405

**Published:** 2022-12-02

**Authors:** Yang Chen, Siyuan Zhang, Lihao Xu

**Affiliations:** School of Digital Media and Art Design, Hangzhou Dianzi University, Hangzhou 310018, China

**Keywords:** spectral reflectance reconstruction, metameric black, organ tissue

## Abstract

In this study, a new approach is proposed for the restoration of reflectance information on organ samples using a commercial camera. This novel approach is comprised of three stages. In the first stage, a color clustering method is utilized to extract the representative colors of the organ samples as well as their corresponding spectral reflectance. In the second stage, the spectral reflectance is decomposed into two separate parts, i.e., the fundamental stimulus spectrum and the metameric black following the matrix-R theory, and the latter is further utilized to form a look-up table (LUT) via a lattice regression model. Finally, the reflectance information can be easily retrieved by referring to the newly built LUT. The performance of the proposed method was investigated, along with that of six other commonly adopted methods, through a physical experiment using real, measured organ samples. The results demonstrate that the proposed method outperformed all the other methods in terms of both colorimetric and spectral metrics, indicating that it is a promising strategy for organ sample reflectance restoration.

## 1. Introduction

Color is crucial to defining the appearance of an object, and is determined by the joint effects of the light source, the object surface, and the human eye. This is to say, the surface color changes when illuminated under different illuminants. So, color alone cannot be thought of as a natural property of an object. Instead, its spectral reflectance, which can be thought of as its “footprint,” should be used to describe the optical property of an object’s surface. 

With the development of multispectral and hyperspectral imaging technology, spectral information has been widely used in a variety of industries, such as textile printing, museum archiving, and biomedical imaging, especially for disease diagnosis and image-guided surgery. The traditional way to obtain the spectral reflectance of an object is by using a multispectral imaging system (MSIS). However, such a device usually has the drawback of being expensive and time-consuming, making it hard to use in real time. Recently, research has been focused on commercial digital cameras to recover the reflectance information from the camera responses [1]. This is encouraging because digital cameras are becoming more portable, rapid, and high-resolution. Therefore, it is of the utmost importance to develop an algorithm that reliably recovers reflectance information from camera responses.

However, recovering high-dimensional spectral information from the low-dimensional camera responses is an underdetermined problem to, indicating that no routine procedure exists [2]. Over the past few decades, numerous approaches have been attempted to address this difficulty, including Wiener estimation, pseudo-inverse estimation, the matrix R method, Principal Component Analysis (PCA), Independent Component Analysis (ICA), and other techniques [3]. These techniques, however, sometimes lack accuracy and fall short of the strict requirements of practical applications. Agahian et al. [4] investigated the weighting factors of the PCA basis vectors, adopting the Euclidean distance in CIELAB color space to determine the weights. The resultant spectra showed considerable improvements in terms of both spectral and chromaticity accuracy in comparison to those obtained from the standard PCA method. Similarly, Xiao et al. [5] developed a polynomial model to determine the weighting factors of the PCA basis vectors, achieving a color difference of less than 3 CIEDE2000 units when applied to skin colors. Amiri et al. [6] proposed a method to perform Weighted Least Square (WLS) regression on the polynomial extension of the sample response matrix in a global weighting form. Theoretically, these strategies are designed to lower the dimension of the reflectance space by using fewer basis vectors. As a result, it is appropriate if the samples contain similar components or if the dimension of the reflectance space is relatively small.

In recent years, regression-based linear and nonlinear models have become more popular. Heikkinen et al. [7] proposed a kernel ridge regression (KRR) method for spectral reflectance, which nonlinearly transforms low-dimensional camera response into high-dimensional feature space and conducts regularized least squares regression of reflectance data in the feature space. Compared to the kernel model with the Gaussian kernel [8] that had already been studied, the results suggest that a link function and a model with a Matérn kernel reduce spectral errors. Shen et al. [9] proposed a spectral reconstruction method based on partial least squares regression(PLS) that extends camera responses by high-order polynomials to deal with nonlinearity and reduces overfitting by using partial least squares regression. Experiment results showed that the method outperformed Wiener estimation and ordinary polynomial regression and that it was comparable to polynomial regression with regularization. A local linear model based on regularization was put forth by Zhang et al. [10], which located the first k samples from the training dataset that were closest to the test sample and used a regularized regression approach to determine their weights. As stated, the model accuracy on the Munsell dataset was superior to other widely used techniques. Similarly, Li et al. [11] proposed a method for spectral reconstruction based on locally linear approximation, which used the optimal weight coefficients of k neighbors in the tristimulus value space to linearly fit spectral reflectance in the spectral space. Experimental results indicated that the method outperformed other competitors in terms of both accuracy and stability for spectral reconstruction. A locally weighted linear regression method was put forward by Liang et al. [12] that employed neighbor colors to establish a local weight matrix to determine the optimal transformation matrix for each test sample. This method was found to give the best performance on the Munsell dataset among all the methods investigated. Later, Liang et al. [13] replaced the original weighting method by adopting an adaptive local weighted linear regression method based on the Gaussian weighting function, which gave the best accuracy on both a standard color chart and a set of textile samples compared with other competitive methods. A nonlinear approach based on kernel partial least squares was proposed by Xiao et al. [14]. It performed spectral reconstruction from nine-channel camera responses using a kernel function and partial least squares. The experimental results showed that the model accuracy on the Munsell and IT8.7/3 datasets was either superior to or equal to that of other methods. Wang et al. [3] proposed a successively weighted nonlinear regression method to estimate spectral reflectance. The distinctive aspect of this approach was the gradual weighting of chromatic aberration, which lowered spectral inaccuracy. As more local samples are taken into consideration, it is anticipated that such regression-based linear and nonlinear models could provide superior performance. However, those methods typically require more training samples and higher computing costs compared to the traditional, basis-based methods.

Due to the proliferation of hyperspectral datasets, deep learning and shallow learning techniques using sparse coding have received a lot of attention. Arad et al. [15] described a method that adopts sparse encoding and dictionary learning to recover the spectral information from a commercial RGB camera. His method was further refined by Aeschbacher et al. [16] by computing a sparse dictionary that contains the corresponding low and high spectral resolution atoms and calculating the reflectance information using the nearest neighbor colors of the anchor. Then, Lin et al. [17] extended the work of Aeschbacher et al. by adopting the nearest neighbors in the spectrum domain instead of the RGB color space domain. The experimental results validated the superiority of these learning-based methods. However, such methods require a considerable quantity of training data and a high computing cost to achieve good accuracy. These conditions are hard to satisfy in some specific fields, such as real-time biomedical imaging, especially for disease diagnosis and image-guided surgery.

In view of the above problems, a novel approach is proposed in this study to reconstruct the spectral reflectance of tissue samples based on the *matrix-R* theory and lattice regression. The whole workflow consisted of three stages. In the first stage, the color clustering algorithm was applied to obtain the representative colors of the organ samples and their corresponding reflectance information. In the second stage, the spectral reflectance was decomposed into the fundamental stimulus spectrum and the metameric black, using the matrix-R theory. A look-up table (LUT) was established using lattice regression to estimate the metameric black, and the fundamental stimulus spectrum could be directly obtained from the spectral sensitivity functions (SSFs) of the camera. In the final stage, the newly established LUT was adopted to perform interpolation to reconstruct the metameric black for each test sample. This was then combined with the fundamental stimulus spectrum to form the final output spectral reflectance. The current experimental results confirmed the superiority of the proposed method in terms of both colorimetric and spectral metrics on organ samples.

## 2. Methods

### 2.1. Spectral Imaging Model

Similar to the imaging principle of human eyes, the responses of a three-channel camera depend on the Spectral Power Distribution (SPD) of the light source s(λ), the surface reflectance r(λ), the camera sensitivity functions $c_{k}\left(\lambda \right)$, and the system noise $n_{k}$. The camera responses $d_{k}$ can be written as the following imaging model, as shown in Equation (1).

(1) $$d_{k}=\int_{\varphi }s\left(\lambda \right)r\left(\lambda \right)c_{k}\left(\lambda \right)d\lambda +n_{k},k\in \left\{R,G,B\right\}$$

where φ represents the visible spectrum. For simplicity, noise is often ignored, i.e., $n_{k}=0$, and the above continuous integration process is often discretized using a matrix form. As a result, the equation can be written as

(2) $$d=A^{T}r$$

where d represents the response vector of the three channels, $A^{T}$ represents the spectral response matrix, which is a multiplication of the SPD of the light source and the SSFs of the camera, while r represents the spectral reflectance of the object.

### 2.2. Matrix R Theory

According to the metameric black hypothesis, the spectral reflectance of an object can be described as having two components: the fundamental stimulus spectrum and the metameric black [18]. The former contributes to camera responses, and the latter produces zero responses, which is the cause of metamerism [19]. Based on this hypothesis, Cohen and Kappauf [20] developed the *matrix R theory*, a mathematical technique for decomposing the reflectance of a color stimulus into the fundamental spectrum and its corresponding metameric black. A brief overview of this theory is provided below. 

The projection matrix of the camera space is defined as:

(3) $$R=A{\left(A^{T}A\right)}^{-1}A^{T}$$

where *A* is the same as in Equation (2). The fundamental stimulus spectrum $r^{\prime }$, is obtained by projecting the spectral reflectance r onto *R*, namely $r^{\prime }=Rr$. The projection matrix guarantees that the spectral reflectance r and the fundamental stimulus spectrum $r^{\prime }$ produce the same camera response. The difference between these two spectra can be considered as the metameric black b,

(4) $$b=r-r^{\prime }=\left(I-R\right)r$$

where I represents the identity matrix. Equation (4) suggests that the metameric black b yields *zero* camera responses.

The most important part of the *matrix R theory* is that the fundamental stimulus spectrum $r^{\prime }$ can be accurately obtained from the camera responses,

(5) $$r^{\prime }=Rr=A{\left(A^{T}A\right)}^{-1}A^{T}r=A{\left(A^{T}A\right)}^{-1}d$$

As a result, the estimation of the spectral reflectance r now turns to the estimation of the metameric black. This can be considered the highlight of this theory, suggesting that the recovered reflectance will reproduce the same camera response as the correct input RGB. This ensures that the reproduction image generated using the recovered reflectance information is the same as the input ground truth image, and thus is often referred to as the “physical plausibility property” in other studies [21].

### 2.3. Lattice-Based Regression

**Figure 1:**
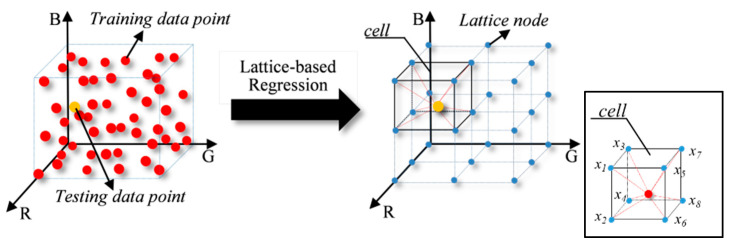
An example of the lattice-based regression LUT. The red point represent the RGB training data points, the yellow point represents an RGB testing data point, and the blue points represent the LUT data points, that is, the lattice nodes.

As previously stated, the main goal of spectral recovery is to estimate the spectral reflectance r, or more specifically, the metameric black, from the responses of the camera. This key component in this paper was accomplished by referring to a LUT established by lattice regression. The concept of lattice regression is illustrated in Figure 1. As demonstrated, the RGB training data are represented as red points, each of which corresponds to a distinct reflectance. The yellow dot denotes a test sample point that needs to be derived from the surrounding training samples. These training samples are typically scattered unevenly, so different weights should be provided to each neighbor color based on their proximity to the test sample point. However, this strategy is empirical and sometimes fails when there are not enough training data points. As a result, it is preferred if all the data points are evenly distributed, as depicted in Figure 1 (right) using blue dots, so that the estimation can be completed in the cell containing that test sample point. In other words, if the reflectance $z_{j}$ corresponding to the lattice node $x_{j}$ can be accurately predicted, the spectral recovery problem can be efficiently resolved via a regular interpolation algorithm. In this study, the common cubic interpolation method was adopted as an example.

This LUT can be constructed using lattice regression with the training dataset. Assume there are ***n*** training sample pairs $\left\{d_{i},r_{i}\right\}$, i=1,…,n, where $d_{i}\in {ℛ}^{3}$ represents *i*th the camera responses (RGB), $r_{i}\;\in {ℛ}^{t}$ represents its corresponding spectral reflectance and t is the dimension of the reflectance. For any sample pair $\left\{d_{i},r_{i}\right\}$, a cell can be easily located within the LUT and its vertices can be utilized to represent the data point $d_{i}$.

(6) $$d_{i}=\sum_{j=1}^{m}w_{i,j}x_{j},\;\sum_{j=1}^{m}w_{i,j}=1$$

where $x_{j}\in {ℛ}^{3}$ represents the camera responses of the *j*th lattice node and $w_{i,j}$ is the ith–jth element of matrix $w\in {\left[0,1\right]}^{n*m}$, representing the weighting factor of each lattice node. The set of weights $\left\{w_{i,j}\right\}$ is determined by the spatial relationships between the training data point $d_{i}$ and the lattice nodes. It should be noted that $w_{i,j}$ is not *zero* only for the cell vertices. For nodes outside the cell, $w_{i,j}$ is kept at *zero*.

Therefore, the corresponding reflectance $r_{i}$ can be estimated as

(7) $${\hat{r}}_{i}=\sum_{j=1}^{m}w_{i,j}z_{j}$$

where $z_{j}$ represents the reflectance of the *j*th lattice node. If we denote the *k*th dimension of $z_{j}$ as $y_{j}$, the corresponding regression error for all training samples in the *kth* dimension can then be written as Equation (8):

(8) $$\sum_{i=1}^{n}{\left({\hat{s}}_{i}-s_{i}\right)}^{2}=\sum_{i=1}^{n}{\left(\left(\sum_{j=1}^{m}w_{i,j}y_{j}\right)-s_{i}\right)}^{2}$$

where the set of $\{y_{j}\},j=1,\ldots ,m$, is the reflectance value of the *kth* dimension associated with the *j*th lattice node. The set of $\{s_{i}\},i=1,\ldots ,n$, is the reflectance value of the *kth* dimension associated with the *i*th training sample. Therefore, the goal of lattice regression is to minimize the Equation (8), as shown in Equation (9):

(9) $$\hat{y}=arg\underset{y}{\min}\sum_{i=1}^{n}{\left(\left(\sum_{j=1}^{m}w_{i,j}y_{j}\right)-s_{i}\right)}^{2}$$

Equation (9) is underdetermined when there is a cell that contains none of the training data points. In this condition, the solution is not unique, and more constraints should be imposed. In this study, a smoothness term is introduced by utilizing the second-order difference of each dimension, also known as the *Hessian regularizer*. It can be written as Equation (10),

(10) $$\sum_{\begin{array}{c} over\;the\;R,\;G,\\ and\;B\;dimensions \end{array}}\sum_{\begin{array}{c} adjacent\;in\;R,\;G,\\ or\;B\;dimension \end{array}}{\left(\left(y_{h}-y_{j}\right)-\left(y_{j}-y_{l}\right)\right)}^{2}=\sum_{d=1}^{3}\sum {\left(y_{h}-2y_{j}+y_{l}\right)}^{2}$$

Equation (10) represents the penalization of the second-order difference, summed over the three dimensions (R, G, and B). Therefore, the mathematical model of the lattice-based regression is as follows:

(11) $$\hat{y}=arg\underset{y}{\min}\left(\sum_{i=1}^{n}{\left(\left(\sum_{j}^{m}w_{i,j}y_{j}\right)\sum s_{i}\right)}^{2}+\lambda \sum_{d=1}^{3}\sum {\left(y_{h}\sum 2y_{j}+y_{l}\right)}^{2}\right)$$

(12) $$\hat{y}=arg\underset{y}{\min}{\left\|Wy-s\right\|}_{2}^{2}+\lambda y^{T}K_{s}y$$

where $y={\left[y_{1},\ldots ,y_{m}\right]}^{T}$; $s={\left[s_{1},\ldots ,s_{n}\right]}^{T}$; $W\in {\left[0,1\right]}^{n*m}$ denotes the set of weights to interpolate the training data and $K_{s}$ is an *m* by *m* matrix. The role of the regularization parameter λ(>0) is to tradeoff between solving accuracy and smoothness. Equation (12) has a closed-form solution,

(13) $$\hat{y}={\left(W^{T}W+\lambda K_{S}\right)}^{-1}W^{T}s$$

The LUT is obtained by calculating the reflectance values for each dimension of the *m* cell vertices using Equation (13). For any input test point $d^{\prime }$, its corresponding reflectance $\hat{r^{\prime }}$ can be estimated using the newly established LUT following Equation (7). Therefore, the whole process of the lattice-based regression LUT is defined as a mapping relation ***Lattice*(∙)**, which represents the mapping of the input camera responses to the corresponding reflectance (the metameric black). This is written as Equation (14),

(14) $$\hat{r^{\prime }}=\mathrm{Lattice}\left(d^{\prime }\right)$$

### 2.4. Workflow

**Figure 2:**
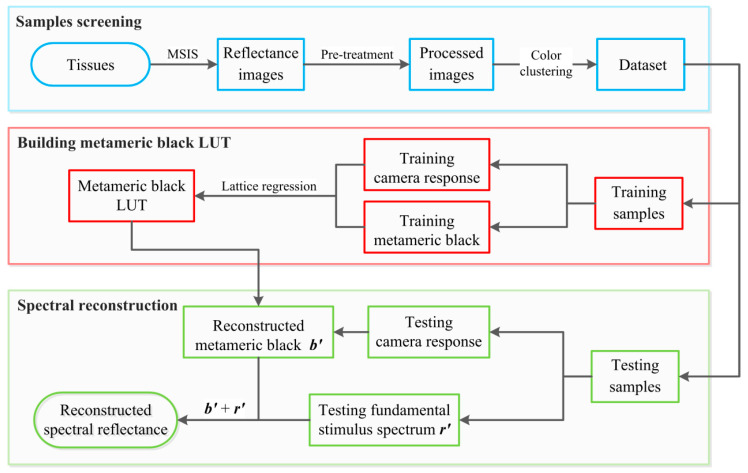
Workflow of the proposed method.

With the methods mentioned above, a new spectral recovery algorithm was proposed in this study to recover the spectral reflectance of organ samples based on matrix-R theory and lattice regression. The workflow is given in Figure 2. Initially, a series of hyperspectral images of organ samples were captured using an MSIS [22]. They were then projected onto the SSFs of a commercial camera to generate their corresponding RGB images. Note that a pre-treatment was implemented to remove the highlights from the surface of the organ samples. The organ image was first transformed into the CIELAB color space. After that, pixels with a lightness value (L*) over a certain threshold were removed. Afterwards, the representative colors of each tissue image were extracted via a color clustering algorithm to form a large dataset, which included both the camera responses (RGB values) and their corresponding reflectance. This dataset was further split into training and testing datasets with the goal of a uniform color distribution.

Following the matrix R theory, the spectral reflectance was decomposed into two parts, i.e., the metameric black and the fundamental stimulus spectrum. An LUT was established between the camera responses and their metameric blacks by lattice regression. For any input test point $d^{\prime }$, the corresponding metameric black $b^{\prime }$ can be obtained by referring to the newly established LUT. A cubic interpolation method was adopted in this study. Meanwhile, the fundamental stimulus spectrum $r^{\prime }$ can be easily retrieved using Equation (15). With these two components available, the final reconstructed spectral reflectance $\hat{r}$ was finally obtained,

(15) $$\hat{r}=r^{\prime }+b^{\prime }=A{\left(A^{T}A\right)}^{-1}d^{\prime }+\mathrm{Lattice}\left(d^{\prime }\right)$$

It should be emphasized that this new algorithm was specially developed for organ samples, which means that metamerism—the phenomenon of lights that elicit the same response from the sensory system but have different power distributions over the sensed spectral segment [23,24]—is a significant problem in this condition. As a result, different camera responses correspond to different recovered spectral reflectances. Moreover, this study followed the same concept of manifold learning [25,26]. In other words, the local linear relationship between samples in low-dimensional RGB space remains the same in high-dimensional reflectance space. These two underlying assumptions form the basis of the proposed approach.

## 3. Experiments

A series of hyperspectral images of organ samples were captured, and were adopted to fully evaluate the performance of the proposed method and six widely used methods.

### 3.1. Acquisition of Samples

**Figure 3:**
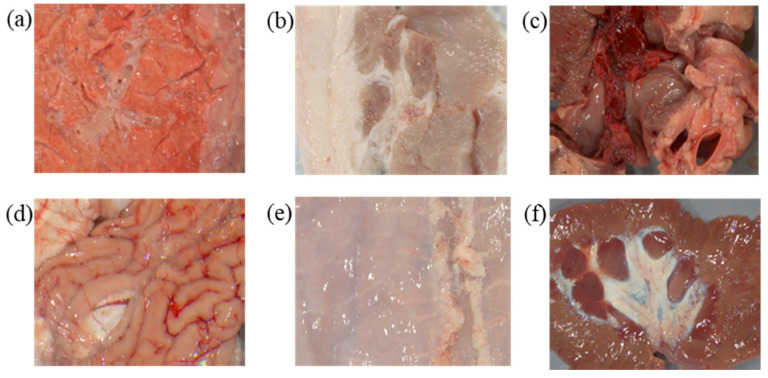
Sample images of organ tissues: (**a**) pig lung, (**b**) pork, (**c**) pig heart, (**d**) pig brain, (**e**) pig bag, and (**f**) pig kidney.

**Figure 4:**
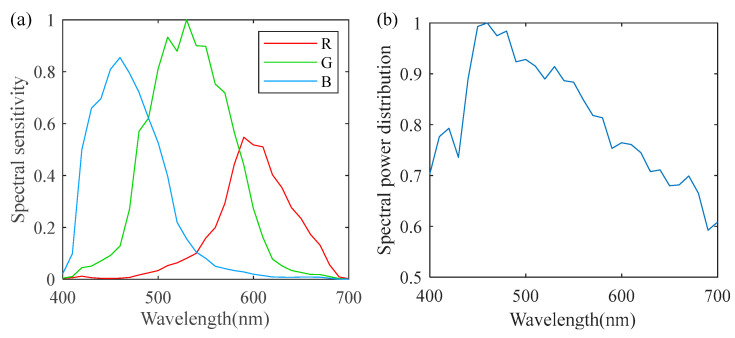
(**a**) The SSFs of the Canon 60D, and (**b**) the D65 illuminant.

A total of 33 reflectance images of biological organs were collected via an MSIS, including a pig’s heart, liver, and so on. Some of them are displayed in Figure 3. The MSIS was composed of an achromatic 14-bit digital camera and 16 narrow band optical filters [22]. The filters had a spectral range of 400 to 700 nm with a 10-nm interval. Its imaging precision was less than a CIELAB unit when applied to fabrics. For the spatial dimension, this MSIS offered a resolution of 1040 by 1392 pixels, which is typical in this kind of application. These hyperspectral images were further projected onto the SSFs of a commercial camera, i.e., the Canon 60D [27], to generate the corresponding RGB images. The SSFs are given in Figure 4a. The illuminant was set at D65 in this conversion. As a result, 33 RGB images were finally constructed.

### 3.2. Samples Screening

**Figure 5:**
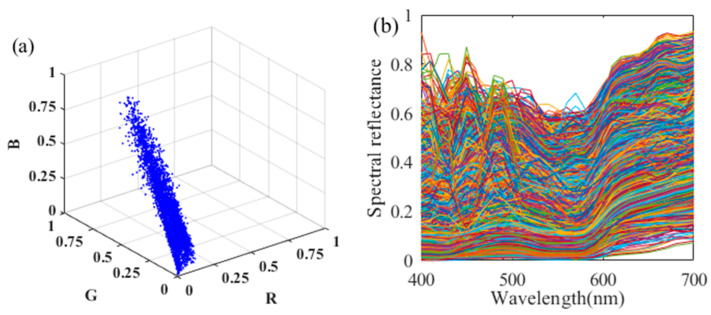
(**a**) Color distribution of the 6529 representative colors and (**b**) their corresponding reflectance curves.

As shown in Figure 3, there is a gray background and a few highlights visible in the captured image. These flaws were eliminated beforehand, leaving only the surface colors of organ samples. After that, the k-means clustering algorithm was applied to those processed images, resulting in *k* color clusters for each image. All the colors within a cluster were then averaged, and they were regarded as the representative colors for each organ image. With such a method, the quantity of color samples was significantly decreased, and the system noise was reduced for each averaged color. In this study, the *k* value was set at 200 for each image, yielding a total of 6600 color samples for all 33 organ images. After that, all those representative colors were carefully inspected, and the defects were manually removed. As a result, 6529 color samples were finally obtained, and were randomly divided into two datasets, i.e., the training dataset and the testing dataset. The camera responses of these colors are shown in Figure 5a, and their corresponding reflectance curves are shown in Figure 5b. The training dataset consisted of 4900 colors, and the remaining 1629 colors were adopted as the testing dataset, with a training-to-testing ratio of approximately 3:1.

### 3.3. Results

Three conventional metrics were adopted in this study to evaluate the model performance, including CIEDE2000 [28] for colorimetric accuracy, root mean square error (RMSE) and goodness-of-fit coefficient (GFC) for spectral accuracy. The calculations of RMSE and GFC are defined in Equation (16).

(16) $$\begin{array}{cc} \mathrm{RMSE}=\sqrt{\frac{1}{t}{\left(\hat{r}-r\right)}^{T}\left(\hat{r}-r\right)} & \mathrm{GFC}=\frac{{\hat{r}}^{T}r}{\left\|{\hat{r}}^{T}\hat{r}\|\cdot \|r^{T}r\right\|} \end{array}$$

where r is the ground truth spectral reflectance; $r^{\prime }$ is the reconstructed spectral reflectance; ***t*** represents the number of sampling points in the visible spectrum from 400 nm to 700 nm, and equaled 31 in this study.

Several frequently used approaches, including Arad’s [15], Connah’s [29], Xiao’s [5], Li’s [11], Agahian’s [4], and Zhao’s [30], were compared to the proposed method. Arad’s method is based on dictionary learning and sparse coding; Connah’s method is based on the least square method of polynomial extension; Xiao’s method is based on polynomial mapping and PCA method; Li’s method is based on the linear approximation method of local k nearest neighbor, and Agahian’s method is based on the global weighted PCA method. Zhao’s method is the traditional R matrix method. The minimal errors (min), mean errors (mean), and maximum errors (max) for all three metrics are summarized in Table 1. The best result is underlined and marked bold. All currently employed approaches, with the exception of Arad’s, were based on the recommendation parameters from the original literature. However, the parameters for Arad’s method were re-optimized to ensure a best performance. The best result was obtained when the sparsity was set to 9 and the dictionary size was set to 40 in this study.

There are two hyper-parameters that should be pre-determined using the proposed method, namely the smoothing coefficient λ and the size of the LUT. An appropriate parameter, λ, can reduce noise and improve the model’s accuracy to a certain extent. Through a pilot test, it was found that a λ value of 0.0015 achieved the best model performance. The size of the LUT, however, does not demonstrate a substantial impact on model performance. Therefore, the size of the LUT was fixed at 25×25×25 in this study to provide a balance between time consumption and reconstruction accuracy.

As shown in Table 1, all methods performed well on the spectral recovery of organ samples, and the proposed approach outperformed all other methods studied. More specifically, the proposed method had a mean RMSE of 0.0159 and a mean CIEDE2000 of 0.59, while the best outcomes of competing methods was 0.0164 (Li) and 0.61 (Li), respectively. It should be noted that a color difference of less than one CIEDE2000 unit is quite small, and observers can barely discern the ground truth color and its reproduction. Additionally, the maximum CIEDE2000 value of the proposed method was greater than 4.5 units, indicating it was not negligible. However, the highest value was typically brought on by random noise during capture and comprises only a small portion of the image, suggesting that human perceptions are not be much affected. Noticeably, Arad’s approach did not work well in this study. This might be due to the relatively small training dataset compared with its original study. More training samples can considerably boost its performance, as demonstrated by earlier studies [16].

A boxplot is a method for graphically demonstrating the locality, spread and skewness groups of numerical data through their quartiles, and was used to demonstrate the performance of different method investigated in this study. The top of the blue rectangular box (box) in a boxplot represents the upper quartile, the bottom represents the lower quartile, and the red line within the box represents the median. The data are more centralized the shorter the box. The horizontal line at the top shows the largest error, while the horizontal line at the bottom represents the minimum error. In general, the maximum (minimum) error is set at 1.5 (−1.5) box sizes (range of the middle quartile) from the third (first) quartile value. The red “+” sign at the top of the graph denotes outliers, which correspond to the higher errors in the data.

**Figure 6:**
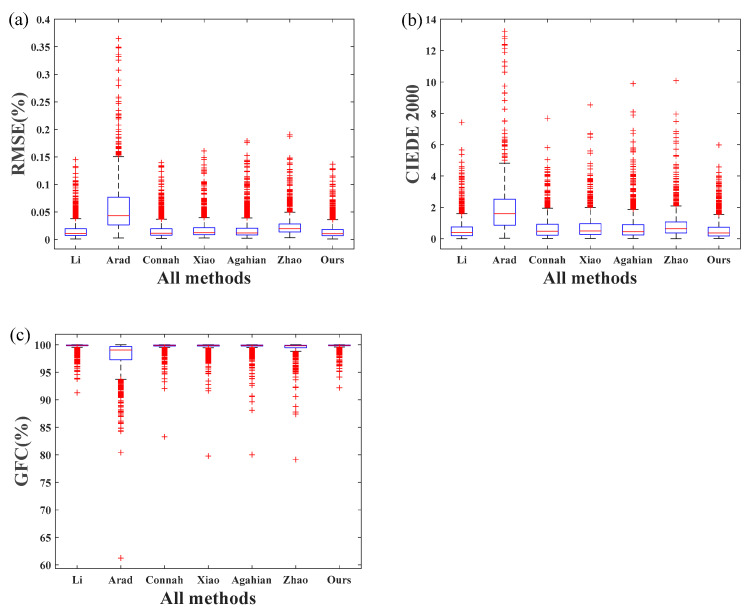
Boxplots of experiment results of (**a**) spectral RMSE error, (**b**) CIEDE2000 color difference, and (**c**) spectral GFC error.

Boxplots were used to demonstrate the distribution of reconstruction errors for all the methods investigated. As illustrated in Figure 6, the proposed method had a more compact error distribution, demonstrating its superiority over all other methods. Li’s method also offered good reconstruction accuracy. Arad’s method performed the worst.

**Figure 7:**
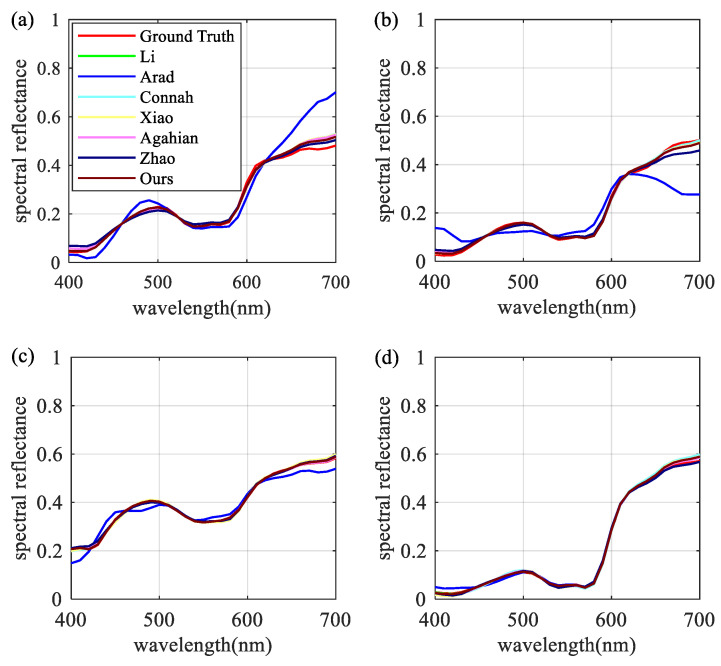
Comparison of spectral reflectance reconstruction results of four randomly selected samples: (**a**) No.76, (**b**) No.576, (**c**) No.708, and (**d**) No.816.

For an intuitive demonstration of model performance, four test samples were randomly selected from the testing datasets. These samples are shown in Figure 7. The majority of the differences were observed in the long wavelength region. Once more, the proposed approach provided the highest accuracy.

### 3.4. Results after Adding Noise

Different amplitudes of additive normally distributed noise were introduced to the camera responses to better mimic real-world settings. The noise had a zero mean and a variance of $\sigma^{2}$ for each individual channel. The signal-to-noise ratio (SNR) was using Equation (17),

(17) $$\mathrm{SNR}=10\log_{10}\left(\frac{{\left\|D\right\|}_{F}^{2}}{{\left\|e\right\|}_{F}^{2}}\right)$$

where D is the noise-free response matrix and e is the noise matrix. ${\left\|\cdot \right\|}_{F}^{2}$ represents the Frobenius norm. The higher the SNR value, the less noise. The accuracy of the spectral reconstruction was examined at three SNR levels: 80, 60, and 40 dB, which can be considered typical in real-world conditions.

Table 2 indicates the superiority of the proposed technique under both conventional and low-noise conditions (SNR of 80 dB and 60 dB). Its performance deteriorated as noise levels rose (SNR of 40 dB). Li’s approach had the smallest color difference for the mean and maximum values, making it the most accurate in terms of colorimetry in the noisy condition. However, the lowest spectral RMSE and GFC errors were still achieved by our proposed method. Such a difference suggests that spectral accuracy may not exactly coincide with colorimetric accuracy.

**Figure 8:**
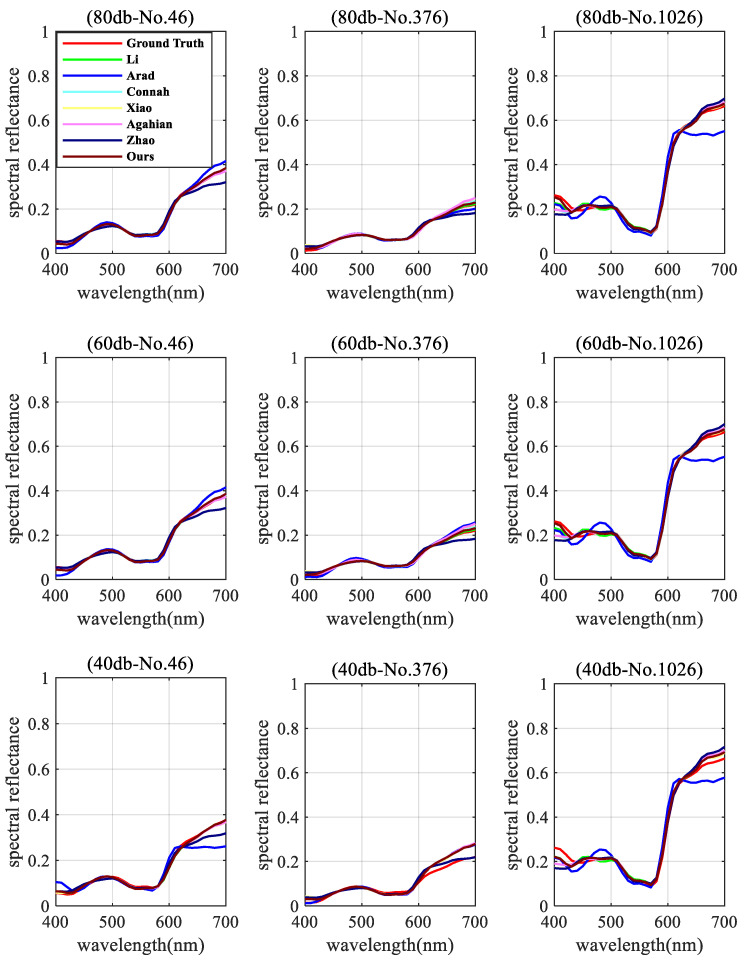
Comparison of simulated spectral reflectance reconstruction with three randomly selected samples (No. 46, No. 376, and No. 1026) at three different noise levels.

In addition, three test samples were randomly selected to demonstrate the model performance at different SNR levels; their results are shown in Figure 8. It is obvious that when noise levels rise, model performance declines.

## 4. Discussions

The main purpose of this study was to reconstruct the spectral reflectance of organ samples using a commercial RGB camera. A large dataset consisting of organ and bio-tissue samples was collected using a MSIS, and a novel spectral reconstruction algorithm was proposed to provide the highest recovery accuracy. Before drawing a final conclusion, factors affecting the model performance were discussed to offer a comprehensive understanding of the proposed method.

More data implies more precise modeling. The core idea of the proposed method is to establish a LUT to transform the camera responses into spectral reflectance using the lattice regression model. Despite the fact that a smoothness constraint is believed to offer accurate estimates for colors outside the color gamut of the training dataset, this assumption is purely theoretical. As a result, the estimation may not be as precise for data points outside the color gamut compared to those within.

**Figure 9:**
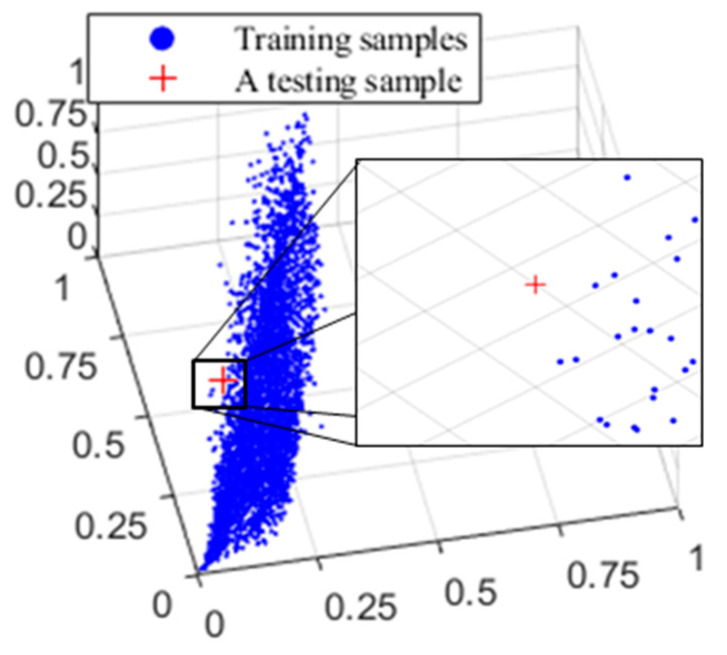
Position of the testing sample with maximum reconstructed color difference.

In addition, the reconstruction accuracy is impacted by the sample distribution. Due to the fact that the reflectance corresponding to each vertex was computed using regression on neighboring training sample points, the estimation was inaccurate for a test color situated in a location with a sparse distribution of training samples. This is inevitable for colors in the boundary regions, which is where the highest colorimetric error occurs, as shown in Table 1. As illustrated in Figure 9, the blue points represent the training samples, while the red “+” represents the test sample with the highest reconstructed color difference. Therefore, estimation is challenging since there are few training samples around the test data point.

Another significant challenge in spectrum recovery is metamerism. It should be mentioned that this study focused mostly on organ samples. This means all the samples had comparable constituents, indicating that metamerism seldom happened. As a result, the LUT was applicable, and the low-dimensional camera responses could be used to represent the high-dimensional reflectance.

It is also worth noting that the proposed method has the potential for use in real-time applications. In contrast to some other time-consuming methods, the proposed algorithm is based on the well-established image processing technology of LUTs. Thus, the training phase, or more precisely, the establishment of the LUT, is the only step that costs time and can be accomplished in only seconds by a typical PC. In a practical application, the RGB image can be converted into a spectral image by employing the LUT in real time.

## 5. Conclusions

In this study, a novel method based on lattice regression and matrix-R theory was proposed for estimating the spectral reflectance of organ samples using camera responses. It was compared with six widely adopted techniques, and the results confirmed its superiority. Moreover, a comprehensive organ dataset made up of 33 hyperspectral images was gathered. The k-means clustering technique was used to analyze these images, and the resulting 6529 organ colors are thought to be representative of organ samples.

## Figures and Tables

**Table 1:** Performances of the proposed method and six existing methods.

Methods	RMSE (%)			CIEDE2000			GFC (%)		
	Min	Mean	Max	Min	Mean	Max	Min	Mean	Max
Arad	0.31	6.41	45.83	0.07	2.02	9.73	77.35	97.11	99.97
Connah	0.16	1.70	13.93	**0.01**	0.71	6.65	83.29	99.75	**100.00**
Xiao	0.27	1.83	16.08	0.03	0.77	7.94	79.79	99.72	**100.00**
Li	**0.08**	1.64	14.47	**0.01**	0.61	5.88	91.31	99.77	**100.00**
Agahian	0.23	1.82	17.91	**0.01**	0.73	9.26	80.04	99.70	**100.00**
Zhao	0.31	2.43	19.08	**0.01**	0.87	8.55	79.14	99.52	99.99
Ours	**0.08**	**1.59**	**13.69**	**0.01**	**0.59**	**4.78**	**92.18**	**99.79**	**100.00**

**Table 2:** Comparison of the performances of the proposed method and six existing methods at three different noise levels.

		RMSE(%)						
SNR	Results	Arad	Connah	Xiao	Li	Agahian	Zhao	Ours
80	min	0.28	0.16	0.27	**0.08**	0.23	0.31	**0.08**
	mean	5.65	1.70	1.83	1.64	1.82	2.43	**1.59**
	max	36.47	13.93	16.08	14.47	17.91	19.09	**13.69**
60	min	0.28	0.16	0.28	0.11	0.23	0.28	**0.10**
	mean	5.65	1.70	1.83	1.64	1.83	2.43	**1.59**
	max	36.49	13.94	16.00	14.47	17.85	19.04	**13.66**
40	min	0.28	0.28	0.39	**0.23**	0.33	0.37	0.27
	mean	5.76	1.87	1.98	1.82	1.99	2.54	**1.79**
	max	35.97	14.13	16.28	14.96	17.94	18.72	**13.42**
		**CIEDE2000**						
**SNR**	**Results**	**Arad**	**Connah**	**Xiao**	**Li**	**Agahian**	**Zhao**	**Ours**
80	min	0.03	**0.01**	0.02	**0.01**	**0.01**	**0.01**	**0.01**
	mean	1.92	0.71	0.77	0.61	0.73	0.87	**0.59**
	max	17.54	6.67	7.94	5.90	9.26	8.55	**4.79**
60	min	0.04	**0.01**	0.04	**0.01**	0.03	0.04	**0.01**
	mean	1.93	0.73	0.78	0.64	0.76	0.89	**0.62**
	max	17.55	9.24	7.97	5.84	9.19	8.48	**4.80**
40	min	0.15	0.07	**0.04**	0.05	0.07	0.10	0.08
	mean	2.35	1.44	1.49	**1.33**	1.55	1.56	1.45
	max	16.60	9.36	11.41	**6.22**	11.42	9.43	9.30
		**GFC(%)**						
**SNR**	**Results**	**Arad**	**Connah**	**Xiao**	**Li**	**Agahian**	**Zhao**	**Ours**
80	min	61.26	83.28	79.78	**91.30**	80.03	79.14	**92.19**
	mean	98.01	99.75	99.72	99.77	99.70	99.52	**99.79**
	max	**100.00**	**100.00**	**100.00**	**100.00**	**100.00**	99.99	**100.00**
60	min	61.25	83.29	79.79	91.36	80.04	79.15	**92.27**
	mean	98.01	99.75	99.72	99.77	99.70	99.52	**99.79**
	max	**100.00**	**100.00**	**100.00**	**100.00**	**100.00**	99.99	**100.00**
40	min	61.19	78.95	78.95	**89.57**	79.45	78.56	88.15
	mean	97.94	99.71	99.71	99.75	99.68	99.50	**99.76**
	max	**100.00**	**100.00**	**100.00**	**100.00**	**100.00**	99.99	**100.00**

## Data Availability

Data underlying the results presented in this paper are not publicly available at this time but may be obtained from the authors upon reasonable request.
